# QIMO: Q-Learning-Based Adaptive Impairment Margin Optimization in DVB-S2X Satellite Communication

**DOI:** 10.3390/s26051462

**Published:** 2026-02-26

**Authors:** Dieter Coppens, Jaron Fontaine, Brecht Reynders, Dieter Duyck, Ingrid Moerman, Eli De Poorter, Adnan Shahid

**Affiliations:** 1 IDLab, Department of Information Technology, Ghent University—imec, Technologiepark-Zwijnaarde 126, 9052 Ghent, Belgium; dieter.coppens@ugent.be (D.C.);; 2 ST Engineering iDirect, Laarstraat 5, 9100 Sint-Niklaas, Belgium

**Keywords:** DVB-S2X, IM margin, MODCOD, ACM, reinforcement learning

## Abstract

Adaptive coding and modulation (ACM) is a key feature in satellite broadcasting; it allows the dynamic selection of modulation and coding (MODCOD) schemes based on channel conditions. The selection is based on the quasi-error-free (QEF) threshold and additional margins. We introduce three distinct types of margins for improved robustness. One of these margins, impairment margin (IM), depends on the nonlinearities of different components in the satellite channel. Current IM selection methods require expert intervention; are costly and prone to errors; and only allow a discrete set of environments. We aim to develop a low-complexity algorithm that converges fast and is quasi-error-free on user traffic due to a non-intrusive exploration method. For this, we propose a Q-learning-based solution that uses passive exploration, with fill frames, to allow error-free IM optimization. Our solution shows a higher average spectrum efficiency compared to expert and default IMs, with fewer low efficiency test cases and more high-efficiency cases.

## 1. Introduction

Satellite communication has become an important part of the current telecommunication infrastructure by providing connectivity to regions lacking terrestrial infrastructure such as ships, aircraft, and remote locations. However, the last-mile connection, from satellite to the user terminals, remains a bottleneck for providing reliable and high-throughput communication. Adaptive coding and modulation (ACM) is a key feature incorporated in the latest satellite broadcasting standard, the digital video broadcasting-satellite second-generation extension (DVB-S2X) [1]. ACM allows the system to dynamically select the best modulation and coding scheme (MODCOD) based on current channel conditions. Selecting an MODCOD is a trade-off between spectral efficiency and robustness. The former is achieved by more complex modulations and encoding schemes with fewer redundant bits, while the latter uses more redundant bits with lower-order modulations. These lower-order modulations are less vulnerable to impairments, noise, and interference. ACM selects an MODCOD according to a channel quality indicator, such as the signal-to-noise ratio (SNR). For this, the algorithm needs the corresponding SNR regions to use a certain MODCOD. These SNR regions are determined based on the quasi-error-free (QEF) threshold of each MODCOD and additional safety margins. The QEF threshold is the SNR at which the MODCOD can be decoded quasi-error-free, which can, for example, be equated with a frame error rate (FER) of $1\times 10^{-3}$. This threshold was determined in a linear additive white Gaussian noise (AWGN) channel and is specified in the DVB-S2X standard. Additional safety margins are added because actual channels are not always linear, and they were added to avoid errors in the case of fading and to protect against wrong measurements. These margins are partly dynamic—based on past channel quality indicators—but mainly static and based on laboratory measurements and selected based on expert knowledge. The chosen MODCOD is the final output of a link-budget calculation ensuring that the received SNR exceeds the QEF threshold plus safety margins for quasi-error-free decoding. Different modulations (QPSK to 256APSK, linear vs. nonlinear variants) exhibit distinct sensitivities to nonlinear distortions and noise, directly affecting required margins. The authors of [2] provide a comprehensive overview of link-margin sensitivity across digital modulations and multi-orbit networks.

This margin selection method has several disadvantages: (1) For each new environment, expert intervention is required, which is costly; (2) the expert configuration is prone to errors, as the expert is human and they do not have perfect knowledge of the system; (3) this method only allows for a few channels/environments between which an expert must choose; in reality, it is more continuous. Errors in the margin configuration can significantly influence the overall performance of the system. Selecting margins that are too high causes suboptimal performance, as a more efficient MODCOD, with higher spectrum efficiency, could have been selected. Selecting margins that are too low causes frame errors, as MODCODs work closer to or below their respective QEF thresholds. These problems show that there is a clear need for a margin optimization algorithm that adaptively selects the margin for each MODCOD such that the throughput is maximized without causing any frame errors and without requiring error-prone human inputs. This need aligns with a broader trend of applying intelligent and learning-based methods to solve complex radio resource management problems in modern communication networks [3]. The solution should fit within the following constraints: (1) Low Complexity: The algorithm should be able to run on the user terminal (limited CPU power), as the perceived channel is different for each. (2) Converges Fast: The performance of the system should quickly come to an efficient solution. (3) Error-Free: The FER should be below $1\times 10^{-3}$ with respect to user traffic.

The primary focus of this paper is to present a Q-learning-based adaptive margin selection algorithm for satellite communication. The main contributions of the paper are the following:

- We propose the use of a non-intrusive exploration method for calculating frame error rates using fill frames (non-user frames) information. This makes our approach unique, as it is the only one that enables system adaptation with zero impact on the user’s experience.
- Introduction of three distinct types of margins for improved robustness: IM, FM, and static margin (SM). The IM accounts for distortion and modulation loss, the FM accounts for predicted future attenuation, and the SM provides additional safety margin.
- Design of a Q-learning-based solution to adapt the margin selection in satellite communication, capable of learning separate IM margins for each MODCOD, to take into account that different MODCODs are affected differently by the channel. The IM is learned at the terminal side; this avoids the need for the signaling of margins in the forward link. Unlike computationally heavy deep Q-learning or LSTM+RL approaches and reactive single-margin methods, QIMO employs lightweight tabular Q-learning that enables fast, MODCOD-specific adaptation at the terminal with zero impact on user traffic.
- Comparison of Q-learning solutions with default and expert margins.

The remainder of this paper is organized as follows. Section 2 discusses the related work for both MODCOD selection/margin selection in satellite networks and the use of Q-learning in satellite networks. In Section 3, the margin selection problem and system model are described. Section 4 describes the proposed Q-learning-based solution in detail. Next, Section 5 describes the evaluation setup, test scenarios, and criteria. Section 6 discusses the performance of the developed algorithm in the different test scenarios. Finally, Section 7 concludes this paper.

## 2. Related Work

A summary of the related work is given in Table 1. Using fixed margins for ACM has been most widely investigated and adopted. In [4], fixed margins to account for fades were proposed, and the authors of [5] showed the trade-off between ACM margin and spectral efficiency, determining an optimal margin (for a single fixed margin).

The related work on solutions to adaptively improve the performance of ACM systems with a fixed margin has several approaches, which can be divided into distinct categories based on the proposed methods.

### 2.1. Reactive Margin-Based Approaches

The authors of [6] propose a scheme to adapt a margin to the current channel conditions. The proposed scheme uses the same margin for all MODCODs, which has the downside of not being valid when there is a switch between MODCODs. Also, the approach constantly lowers the margin until a packet error occurs, which means there will continuously be some errors in the user’s traffic. The authors of [7] avoids the problem of constantly lowering the margin using an adaptive margin derived dynamically during a fade event based on the current fade slope. However, only one margin is used for all MODCODs. This “one-size-fits-all” approach forces a compromise, as the margin must be conservative enough for the most sensitive MODCOD. In summary, these reactive approaches, while dynamic, are fundamentally limited by their use of a single, non-specific margin for all MOCODs.

### 2.2. Machine Learning for Margin Adaptation

The authors of [8] proposed adaptive backoff margins, where MODCOD selection is integrated with a margin obtained by a stochastic gradient descent that uses the decoding history. Like the reactive methods, this approach is still limited to optimizing a single, shared margin. In [9], link adaptation for mobile satellite links was proposed, and nonlinear power amplifiers were considered. The authors proposed adding a margin to the estimated SNR value based on the input power and channel state. However, the margin must be determined beforehand and numerically for all input powers and channel states. While this study considers nonlinear effects, there is still the drawback of only a single margin for all MODCODs, and the margin needs to be determined beforehand and numerically. The authors of [10] proposed using Long-Short Term Memory (LSTM) networks to predict the MODCOD scheme for the next step. They then used reinforcement learning to determine the margin to be added to the forecasted SNR, resulting in the final SNR threshold used for MODCOD selection in ACM. Deep Q-learning and deep deterministic policy gradients are used to determine the margin. Despite using advanced forecasting and reinforcement learning models, the final output is still a single margin value, which does not account for the individual performance characteristics of each MODCOD. Ultimately, although these methods employ sophisticated machine learning for dynamic adaptation, they still converge on a single, shared margin, failing to address the distinct performance characteristics of individual MODCODs.

### 2.3. Direct MODCOD Selection Using Machine Learning

A different approach in the literature bypasses the concept of a margin entirely, using ML to select the transmission parameters directly. For example, in [11], the use of deep Q-learning was proposed for radio resource allocation. Deep Q-learning selects the best configuration parameters (modulation scheme and order, encoding rate, symbol energy and rate, bandwidth, and roll-off factor) to optimize multiple objectives like the error rate, throughput, spectral efficiency, and power efficiency. This has an important disadvantage. Deep Q-learning uses neural networks that are trained using backpropagation, which requires intensive computational resources that are not available in many satellite communication terminals. Other research highlights the trend in applying complex neural networks to different parts of the DVB-S2X communication chain. For instance, the work in [12] applies a convolutional neural network (CNN) to the receiver-side problem of the blind MODCOD identification. While this addresses a different task, it identifies the MODCOD without explicit signaling, rather than selecting it at the transmitter. This further demonstrates the reliance on computationally demanding models for advanced functionalities. This stands in contrast to our approach, which is designed to be lightweight. Therefore, while directly selecting the MODCOD with machine learning circumvents the margin issue, the surveyed approaches rely on computationally expensive models that are unsuitable for deployment on typical satellite hardware.

In conclusion, although various approaches have been proposed to improve ACM, the state of the art is largely constrained by two drawbacks: the reliance on a single adaptive margin for all MODCODs and the high computational cost of direct ML selection methods. Unlike deep Q-learning or LSTM+RL approaches [10,11], which rely on large neural networks and backpropagation (unsuitable for terminal hardware), QIMO employs lightweight tabular Q-learning (66 MODCOD states × 40 IM actions = 2640 entries, 10–20 kB memory). Updates are O(1) scalar operations after frame accumulation (Equation (6)), with fast initial convergence followed by refinement (minutes to hours in operational traffic), while ensuring zero user-data errors through passive fill-frame exploration. In contrast, reactive single-margin methods [6,7] cause ongoing user errors, and forecasting-based RL [10] still outputs a single shared margin. The MODCOD-specific margin, as proposed in this paper, is an important benefit, as each MODCOD can be affected by the channel differently. By using MODCOD-specific margins, this can be accounted for, while a single margin will always have to be high enough for the worst-case MODCOD. Furthermore, our approach is unique in its use of the fill-frame FER, allowing for adaptation without inducing errors in user data.

## 3. System Model and Problem Formulation

### 3.1. Traditional System Overview

**Figure 1:**
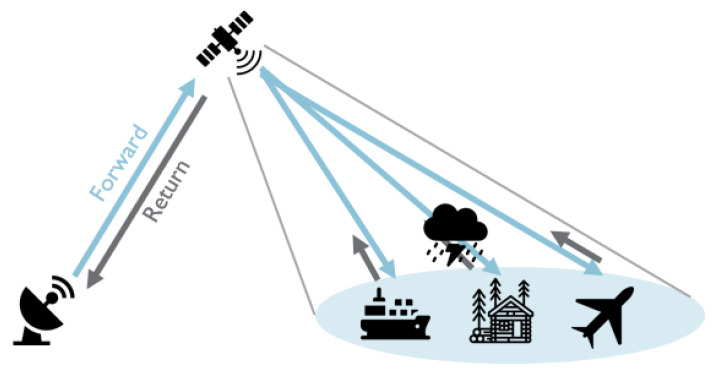
Illustrating satellite communication: the forward link, from hub to user terminals (blue arrows), and return link, from user terminals back to hub (gray arrows).

**Figure 2:**
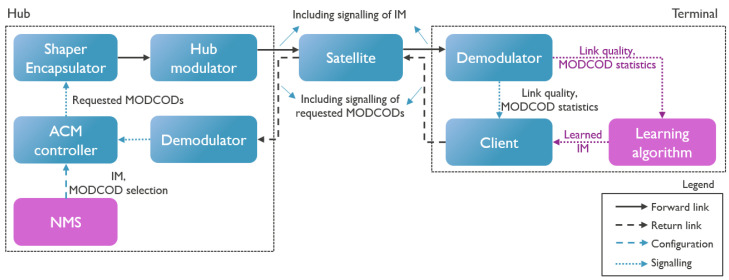
Overview of satellite communication system with emphasis on the essential subcomponents for ACM; the signalling information in each link is indicated. The position of the learning algorithm in this system and the new flow of information are indicated in purple.

A typical satellite network is illustrated in Figure 1, where we visualize the forward and return link. In this paper, we focus on the forward link; however, the results are also applicable for single-channel per-carrier (SCPC) return links. In the forward link, DVB-S2 (X (Annex-M)) is used, meaning that all terminals in a satnet listen to a common single (virtual) carrier. This system relies on a technology known as timeslicing, where a ’virtual carrier’ represents the collection of frames assigned to a specific satellite network within a single, wideband physical carrier. This approach enables the use of more affordable terminals such that low-cost receivers with limited decoder throughput can limit themselves to only decode that subset. Furthermore, this single-carrier architecture has been shown to be more efficient than transmitting over multiple separate physical carriers [13]. The return link is transparent; therefore, there is no loss of generality. In this work, we use an SCPC return link. Figure 2 presents a more detailed illustration of the system, with emphasis on the essential subcomponents for ACM. On the left-hand side is the hub containing the ACM controller. The ACM controller receives its settings, including the available MODCODs and their respective margins, from the network management system (NMS). These settings are signaled to the ACM client that resides in the terminal shown on the right-hand side. The ACM client knows the QEF thresholds of its demodulator for all the MODCODs and monitors the link quality (e.g., SNR). Based on the available information, it determines the most efficient future MODCOD that can be received without error by its demodulator. This selection is then fed back to the ACM controller in the hub. Upon arrival at the ACM controller, this MODCOD is used to fill the baseband frames with the selected MODCOD.

Determining the most efficient future MODCOD is based on the QEF thresholds and additional margins. We distinguish three different types of margins: IM, FM, and SM. The FM is a dynamic margin used to compensate for fades, and it is calculated based on past channel quality indicators, such as the SNR. Many papers have investigated fade detection and prediction such as [14,15,16,17,18], so we will not go into detail about this margin. The IM, on the other hand, depends on the nonlinearities of the different components in the satellite channel, specific user terminals in the network, and channel conditions from the hub to the terminal. The rationale behind the IM is to keep the error boundary of $QEF_{threshold}+IM$ at $1\times 10^{-3}$. For example, in a linear channel, the QEF alone has an error boundary at $1\times 10^{-3}$, which means that the ideal IMs are 0. In nonlinear channels, the QEF alone is not enough, and IMs higher than 0 are necessary to reach the $1\times 10^{-3}$ error boundary. Finally, the SM is added to ensure safety against incorrect measurements and sudden fades.

**Figure 3:**
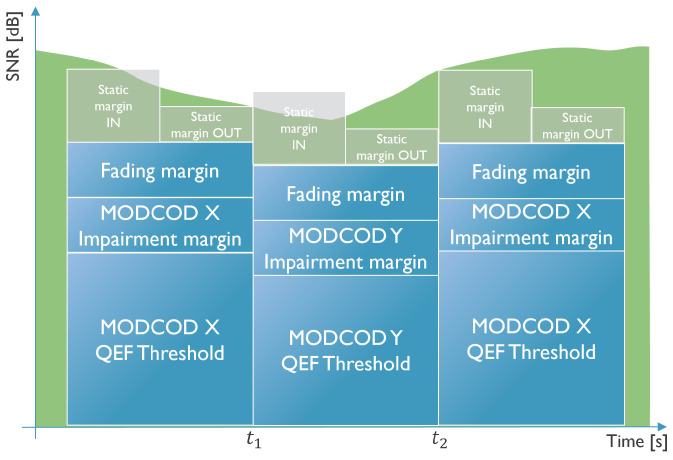
Illustration of MODCOD selection based on SNR (in green, changing over time) and the combination of QEF threshold and additional margins (QEF, IM and fading margins in blue, static margins in gray).

The QEF thresholds are stored locally at the client, FM is calculated locally, and the additional margins (IM and SM) are received through signaling. In Figure 3, the decision between MODCOD *X* and *Y* is shown with a changing SNR over time. At $t_{1}$, $SNR<QEF_{threshold_{X}}+IM_{X}+FM+SM_{OUT}$, which indicates that the SNR is becoming too low to keep using MODCOD *X*. This causes a switch to MODCOD *Y*, which is a more robust MODCOD but has lower spectrum efficiency. At $t_{2}$, the SNR has increased, and $SNR>QEF_{threshold_{X}}+IM_{X}+FM+SM_{IN}$, indicating that the SNR has increased enough to switch back to the more efficient MODCOD *X*. The SM clearly demonstrates some hysteresis to avoid excessive signaling in the return link, allowing for a more stable ACM system.

### 3.2. Problem Description

To achieve high efficiency, the IMs are pivotal: They need to be sufficiently high to guarantee error-free communication while also being sufficiently low to achieve the desired high efficiency. Therefore, an extensive measurement campaign in a lab environment was carried out to measure the QEF threshold and, as a result, the IM in a set of linear and nonlinear scenarios. The aim of this campaign was to correlate the observed nonlinearities in actual setups with one of the test scenarios, and then, we configure the corresponding measured margins as the optimal margins.

The current method of configuring IMs relies on expert intervention and measured data. This method has several drawbacks: (1) It is costly, as it requires human expertise for each new environment; (2) it is error-prone, as the expert can make mistakes due to lack of information; (3) it is discrete and static, as it only allows for a finite set of channels/environments between which an expert must choose—in reality, the channel variations are more continuous; (4) it is not user-friendly. Errors in the IM configuration can significantly affect the overall performance of the ACM system. The problem we aim to address in this research is how to design an adaptive and intelligent method of configuring IMs for ACM systems that can cope with different and varying channel conditions without requiring expert intervention. The algorithm should satisfy the following criteria: (1) Low Complexity: It should be executable on the user terminal with limited CPU resources and account for varying channel conditions. (2) Fast convergence, it should rapidly reach an optimal solution. (3) Acceptable FER: It should not exceed the threshold of $1\times 10^{-3}$ for user data.

The goal is to reliably transmit as much data as possible. This is depicted below:

Maximize:

(1) $$\begin{array}{c} P=\left(1-FER\right)*\bar{\eta } \end{array}$$

where we combine the efficiency $\bar{\eta }$ with the frame error ratio. One could read this as the amount of correctly received bits per unit bandwidth.

In more detail, we have

(2) $$\begin{array}{cc} \bar{\eta } & =\int_{SNR}\eta \left(snr\right)\\ \eta \left(snr\right) & =\max_{m\in M}\left\{\eta_{m}:QEF_{m}+IM_{m}<=snr\right\}\\ FER_{M} & <FER_{max} \end{array}\;$$

These equations define the average spectral efficiency $\bar{\eta }$ obtained by integrating (or averaging) the optimal spectral efficiency η over the SNR distribution under testing.

## 4. Proposed Methodology

In this paper, we propose Q-learning for adaptive IM optimization (QIMO). Figure 2 illustrates where the learning algorithm will be positioned in the complete system; it is part of the terminal because the link quality and MODCOD statistics are readily available there, and in this way, each separate hub–terminal combination can learn the IMs individually for the specific link between them. This has the added benefit that the IMs no longer need to be signaled in the forward link, as they are learned at the terminal itself.

### 4.1. Fill Frames

During demodulation, we collect the following information for each of the MODCODs: (a) the number of frames transmitted during a certain time interval, (b) the number of frames unsuccessfully received during a certain time interval, and (c) an estimate of the SNR at which this reception occurred. As mentioned in the introduction, all optimization approaches require trying out possible solutions that could potentially cause frame errors in user data. This means that these approaches violate the constraint on the FER of the user data, as it would be detrimental for the user data when the IM is too low. If no other sources of information than statistics on user data are available, finding the optimal margins would not be possible without frame errors in the user data.

To overcome this, we propose “fill frames”. Fill frames are measurement frames filled with data that are being decoded with a certain MODCOD. They are only inserted in the (virtual) carrier when there is spare capacity. Frame errors are allowed on these fill frames because they do not affect the performance experienced by the user. The statistics of these fill frames provide the necessary data for optimization. The fill frames consist of a sequence of carefully selected MODCODs such that all modulation orders and linear and nonlinear MODCODs are used. More importantly, the QEF-thresholds of these MODCODs span the entire range of available SNRs. Currently, in the modulator, 32 of the total 66 MODCODs are selected for the fill frames. Selecting these fill-frame MODCODs carefully is important, as these are the only MODCODs that directly learn the IM from fill-frame data. The chosen set of 32 provides representative coverage across modulation orders and DVB-S2/S2X linear and nonlinear clusters, while maintaining sufficient frame accumulation per MODCOD for timely convergence. This reflects a trade-off between learning granularity and convergence speed, where increasing the number of fill-frame MODCODs would improve direct resolution but reduce the number of frames per MODCOD, as the total number of insertable fill frames is limited.

Note that when a link is completely filled, a typical satellite network still exhibits a variety of MODCODs across different terminals. These user frames can also be used for learning, meaning that fill frames are not strictly required for adaptation but rather accelerate convergence and guarantee learning opportunities when spare capacity is available.

Table 2 gives an example of a statistics list that will be received by the proposed learning algorithm at each time step. It shows one MODCOD with significantly more frames; this is the MODCOD used for the transmission of user data. The other MODCODs have a low number of frames, as these MODCODs are part of the fill frame’s MODCOD set. In deployed systems, samples are obtained passively from the demodulator’s per-MODCOD statistics (see Table 2) on both fill frames (inserted opportunistically on spare capacity) and user frames (variety of MODCODs with respect to different terminals always present, even under high load). Using this method, no manual labeling is required, as the algorithm runs fully self-supervised and online.

### 4.2. Q-Learning

**Figure 4:**
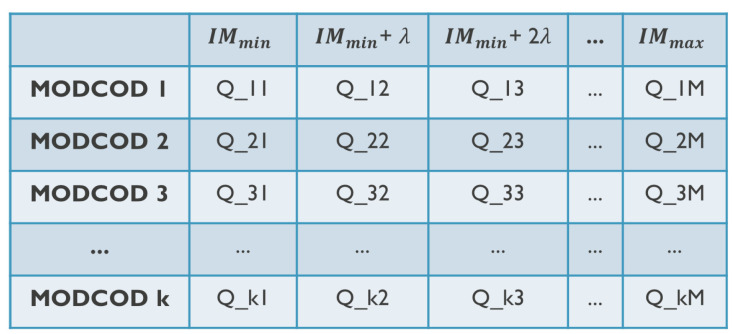
Illustration of the Q-table; the rows represent different MODCODs, and the columns show IM.

**Figure 5:**
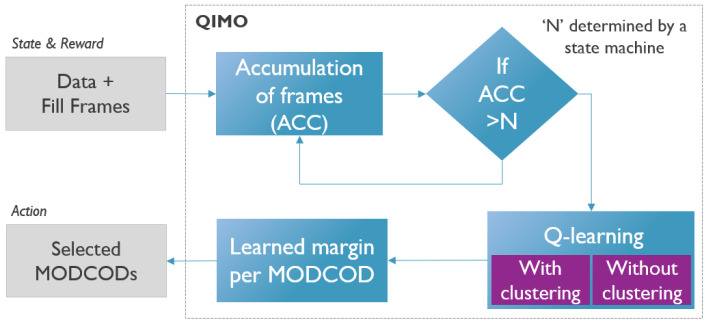
Illustration of different steps that are part of the proposed QIMO solution.

Q-learning is a reinforcement learning (RL) algorithm, a type of machine learning used to train agents to make decisions within a given environment. Based on the actions it takes, the agent obtains rewards or penalties that allow it to gain “experience”. The most important part of the algorithm is the Q-table. Each row in the table represents a state of the system, and each column represents an action. Each value in the table represents the “quality” of a particular state–action pair. A more detailed schematic of the Q-table is given in Figure 4. The algorithm’s objective is to discover a policy that maximizes the cumulative rewards received over time. To apply Q-learning to this problem, we adapted the general Q-learning algorithm in several ways. QIMO is illustrated in Figure 5, with different steps clearly shown.

We have MODCOD as the state and IM as the action. There are 66 MODCODs and, thus, 66 rows in the table. For the columns, the IM had to be discretized. We assume that the minimal IM is −1 dB and the maximal IM is +3 dB. The possible IM values are selected with a step of λ=0.1 dB, resulting in a total of 40 columns. The Q-value thus represents the value of an MODCOD-IM combination.

#### 4.2.1. Reward Function

The reward function is a mapping of a state–action (MODCOD/IM) pair to a numerical value that describes the perceived desirability of that pair based on the received information. Applied here, the reward function needs to describe the value of selecting an IM for an MODCOD. Using the requirements and goals, some assumptions can be made for the design of the reward function:

- Negative reward if FER > $FER_{max}$.
- Positive reward if FER < $FER_{max}$.
- The lower the IM (more efficient), the higher the reward for the same FER.

Using this, the following reward function was constructed:

(3) $$\begin{array}{ccc} \; & R=\; & \left\{\begin{array}{cc} 1+(a\cdot {\left(FER_{max}-\mathrm{FER}\right)}^{b})-c\cdot IM+e & :\mathrm{FER}<FER_{max}\\ 1+\frac{d\cdot (FER_{max}-FER)))}{f}-c\cdot IM-e\;\;\;\;\;\; & :\mathrm{FER}>FER_{max} \end{array}\right. \end{array}\;$$

Here, $a=5/FER_{max}$, b=3, c=1000, $d=1/FER_{max}$, e=4000 and f=50. The constants were selected via trial and error, guided by a qualitative understanding of the trade-offs between error performance and spectral efficiency following standard RL principles for constrained optimization. The reward strongly penalizes $FER>FER_{max}$ while favoring efficient IMs under acceptable FER levels. Parameters *a* and *d* scale FER sensitivity; *b* controls nonlinear sub-threshold selectivity, favoring very low FERs; *c* penalizes inefficient IMs; *e* creates a strong reward gap between acceptable and violating cases (e=4000 ensures safety); *f* smooths the FER violation penalty slope. The IM penalty (c=1000) maintains efficiency pressure under safe conditions. Across all 13 evaluation scenarios (see Section 6.3), QIMO consistently satisfies $FER\leq 10^{-3}$ while achieving superior or comparable throughput and packet loss.

#### 4.2.2. Action Selection Policy and Exploration

In traditional RL algorithms, there is a fundamental trade-off between exploring the environment and exploiting previously learned knowledge. The aim of the action selection policy is to balance the trade-off between exploitation and exploration. In this problem, high error rates on user data are not allowed, which means that traditional exploration techniques are not applicable here. Another difference is that at every time *t*, the algorithm needs to select the IMs for all MODCODs and not a single “current” MODCOD. This means that our algorithm does not switch from one state to the next based on the action selection. Instead of the traditional exploration method, data from fill frames is used to determine the range of IM values for which the received statistics are valid. This range depends on the SNR, FER, and QEF thresholds. The current IM for which the statistics provide information is given by

(4) IM=SNR−QEF.

If the FER is lower than $FER_{max}$ for the current SNR, we know that FER will also be lower than $FER_{max}$ for higher SNR values. This means that the table can be updated from the current IM to the maximum IM value in the table: $\left[IM,IM_{max}\right]$. If the FER is higher than $FER_{max}$ for the current SNR, we know that FER will also be higher than $FER_{max}$ for lower SNR values. This means that the table can be updated from the minimum IM value in the table to the current IM: $\left[IM_{min},IM\right]$. Exploration in this problem uses the information from non-user data in the fill frames to update the Q-table in several rows (fill-frame MODCODs) and columns (deduced IM range) at each time step.

#### 4.2.3. Updating the Q-Table

The foundation of the Q-learning algorithm is the Bellman equation, which is a value update function using the newly received information and weighted old value:

(5) $$\begin{array}{cc} Q_{new}\left(s_{t},a_{t}\right) & \leftarrow Q_{old}\left(s_{t},a_{t}\right)\\ \; & +\;\alpha \left[R_{t}+\gamma \max_{a}Q\left(s_{t+1},a_{t}\right)-Q_{old}\left(s_{t},a_{t}\right)\right]. \end{array}\;$$

The following parameters are used in the equation:

- $s_{t}$: The state at time *t*.
- $a_{t}$: The IM for which information was received.
- $R_{t}$: Reward received for $a_{t}$ (IM).
- α: Learning rate—this factor determines the weight that is given to the newly acquired information and how much old information can be overridden.
- γ: Discount factor—this factor determines the weight that is given to newly acquired information.

Using this function, the values in the Q-table are filled in. After enough iterations, the values in the table will reflect the quality of the state–action pairs, and the expected value of the total received rewards will be maximized. The reward clearly drives the behavior of the algorithm, making the reward function crucial.

However, this problem does not allow us to determine the next state. Because unlike normal Q-learning, at each time *t*, information is received about different MODCODs (states) and a range of IMs (actions) meaning that this update equation is used several times. Additionally, at each step, the action is selecting the IM for all MODCODs (states), which means there is no clear next state or time dependence between different IMs and MODCODs. This is addressed by deliberately choosing γ=0, updating each MODCOD-IM pair independently. By filling in the MODCOD as the state and IM as the action, the update function simplifies to the following:

(6) $$\begin{array}{cc} Q_{new}\left(M_{m},IM\right) & \leftarrow Q_{old}\left(M_{m},IM\right)\\ \; & +\;\alpha \left[R-Q_{old}\left(M_{m},IM\right)\right]. \end{array}\;$$

### 4.3. Accumulation of Frames

At each time *t*, QIMO receives a list of statistics (as discussed in Section 4.1 and illustrated in Table 2). However, because the number of frames in one list of received statistics is limited (certainly for fill frame MODCODs), it is difficult to draw conclusions about an IM being above or below $FER_{max}$. Therefore, frames are accumulated, and the Q-table is only updated once a defined minimum number of frames is received for an MODCOD at a particular SNR. To limit memory usage, it is important to only store relevant information. For each MODCOD, the SNR values around the QEF threshold of an MODCOD are relevant to update the Q-table. This means that we can use a data structure similar to the Q-table, where instead of a Q-value, the total number of frames and total number of errored frames are saved. Choosing the minimum number of frames before updating the Q-table is an important trade-off in the system. The higher this number, the more accurate the FER we use for the Q table. However, it will take longer for the ML algorithm to converge to the optimal solution. Too low a value might result in an IM update that uses an FER of zero, while the actual FER (when more frames are received) is above $FER_{max}$. This can lead to frame errors in user data. Therefore, it is important to choose a value that is high enough to avoid errors in user data, but not so high that it leads to excessive convergence time.

### 4.4. Interpolation

Only 32 of the total of 66 MODCODs are used in the fill frames. This means that for about 50% of the MODCODs, the algorithm is not able to explore and learn the optimal margins directly on non-user data. The only way to get data for the MODCODs not in the fill frames is via user data; an MODCOD can only be selected when the SNR is above the QEF threshold + IM. This means that using only user data, the learned IM of our algorithm can never be lowered from the initial safe margin. To remedy this, the learned margin on fill frame MODCODs will be interpolated to other non-fill-frame MODCODs. This interpolation of the margin between MODCODs is only valid within certain “clusters” of MODCODs. First, the MODCODs are separated based on modulation order (QPSK, 8PSK, …). Then, within a modulation order (if available) a cluster is split up in the DVB-S2 MODCODs, the DVB-S2X nonlinear MODCODs, and the DVB-S2X linear MODCODs. This is visually depicted in Table 3. Within one cluster, linear interpolation is used to determine the margins of the non-fill frame MODCODs.

Although satellite amplifier distortion is inherently nonlinear, interpolation is restricted to MODCODs within the same modulation order and DVB-S2/S2X linear or nonlinear cluster. Within such clusters, the constellation geometry and peak-to-average-power ratio remain fixed, while performance differences arise mainly from coding rate, which varies smoothly. Thus, interpolation is applied only where impairment margin behavior is expected to vary monotonically.

### 4.5. Overall Algorithm

#### 4.5.1. Initialization

When the algorithm is started, there is no prior knowledge about the channel and its characteristics. Therefore, the Q-table is initialized to “safe margins” that do not result in user error in all possible scenarios. Only the MODCODs in the QPSK cluster have a “safe margin” that is in the Q-table (lower than 3 dB), which is around 1 dB. For these MODCODs, a positive value is filled in the Q-table.

#### 4.5.2. Continuous Learning

During continuous learning, the goal is to learn the optimal margins of the current situation and adapt to changes as quickly as possible. As discussed in Section 4.3, there is convergence time vs. user error trade-off in the algorithm. To try and get around this trade-off and combine fast convergence with the low FER on user frames, the accumulation can be split up into two phases: (1) a fast converge phase and (2) a slower optimization phase. In the fast convergence phase, *N* is low “N-low”, which means that the table will be updated quickly, and convergence is fast. However, this means that there is a higher possibility of errors in user data. To avoid this, extra safety is added on top of the margins selected by the system. For example, our system quickly learns that the margin of an MODCOD is 0.2 dB, but because this margin is learned on a possibly inaccurate FER value, an additional safety margin of 0.3 dB is added to avoid errors in this phase. This will result in lower spectrum efficiency during this phase. After this fast convergence phase, the algorithm proceeds to the slower optimization phase, where the table is only updated when a higher number of frames is received: “N-high”. When the higher number of frames is received, the additional safety margin is removed. These two phases are separate for each cell in the Q-table.

#### 4.5.3. Re-Convergence Signal

To allow the system to quickly reset if the channel suddenly changes, we define a re-convergence signal. This happens when unexpected errors are detected in the system. An unexpected error is defined as receiving high user errors (FER > 0.7) that are unexpected based on the accumulation memory. This indicates that the environment has probably changed, and to avoid many additional user errors, the system needs to restart and re-converge. Therefore, the accumulation memory is wiped, and the Q-table is reset to safe margins.

#### 4.5.4. Complexity and Memory Footprint

QIMO stores a 66 × 40 Q-table (2640 floating-point values, ±10–20 kB RAM) with a similarly sized accumulated (errored) frame table, and it performs simple scalar updates with minimal CPU load on embedded terminal processors. No backpropagation, replay buffer, or additional signaling is needed. This contrasts sharply with resource-intensive deep Q-learning (neural-net inference/training, orders-of-magnitude higher memory/FLOPs), supporting the low-complexity claim and practical deployability on user terminals.

## 5. Evaluation Description

### 5.1. Test Scenarios

In this section, we evaluate the proposed Q-learning solution to assess its performance compared to the expert-based solution. First, the experimental setup is described. Then, we describe how the measurements are performed and the criteria for success, and lastly, we discuss and analyze the results.

### 5.2. Experimental Setup

**Figure 6:**
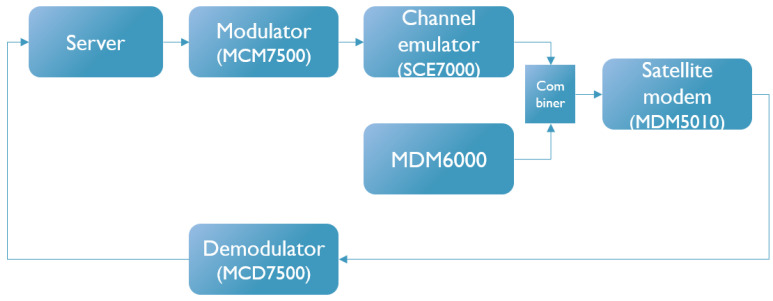
Overview of the test setup used during evaluation.

An overview of the setup used during evaluation is shown in Figure 6. The server controls the system and stores the results of the different tests. It connects to the MCM7500 [19], which is a wideband multi-carrier satellite modulator that is fully compliant with DVB-S2 and DVB-S2X standards [1,20]. To recreate transmission to the satellite and then back to the terminal, an in-house SCE7000 satellite channel emulator is used. It can be used to impair signals with nonlinearity, AWGN, and programmable fade events. The output of the SCE7000 can be combined with the output of the MDM6000 satellite modem to generate a sweeping pure carrier interference in a test scenario. The final signal is received at the MDM5010 high-throughput satellite modem. Together, this forms the forward link. MDM5010 connects to the MDM7500 wideband multi-carrier satellite demodulator to form the return link. No channel emulation is performed here, as it is not relevant to the problem in this research.

The test scenarios are defined and described in Table 4. The channel in a scenario is either linear or nonlinear. When it is nonlinear, the nonlinearity is described based on input back-off (IBO), small signal gain (SSG), amplitude modulation and phase modulation (AM-PM) distortions, and channel compression. Most scenarios have a continuous SNR fade between 8 and 18 dB; three exceptions have a fixed SNR (scenarios 6, 12, and 13). In practice, an expert can make a configuration mistake. To account for this, there are some scenarios where this has occurred (scenario 1 and 7). Each scenario consists of two phases. Phase one is a sort of convergence phase, where, at the start, QIMO begins at the initial safe margins. If the channel is not dynamic, the channel in the second phase is the same as in the first one. If the channel is dynamic, the second phase indicates the start of a sudden change in the channel (scenarios 3, 4, 8, and 9), to which QIMO needs to adapt the previously learned margins from phase one.

### 5.3. Evaluation Criteria

The goals of QIMO are to (1) maximize throughput while (2) not having packet loss and (3) to minimize convergence time. To evaluate these goals, we monitor three metrics in the different test scenarios:

- Total throughput transmitted during 1 min after convergence.
- Total packet loss during 1 min after convergence.
- The time needed to converge.

The throughput and total packet loss monitored can be combined in a single performance metric. This is a value that should indicate the total performance of margins used because it also incorporates the errors made. The way metric $P_{score}$ is calculated is shown in Equation (7) and is equivalent to Equation (1).

(7) $$\begin{array}{ccc} \; & P_{score}=\; & \frac{\#\mathrm{Decoded}\;{\mathrm{Frames}}_{\mathrm{MDM}}}{\#transmitted\;frames_{MCM}}\cdot \frac{\#\mathrm{Transmitted}\;{\mathrm{Bits}}_{\mathrm{MCM}}}{\#\mathrm{Transmitted}\;{\mathrm{Symbols}}_{\mathrm{MCM}}} \end{array}$$

$P_{score}$ gives a single value to the performance of all IMs selected and should be a good estimator for the goal described in Section 3.2 (Equation (1)). Comparing the performance at different SNR values, the spectral efficiency can be plotted for the MODCOD selected at each SNR value.

## 6. Evaluation Results

To evaluate the performance of QIMO, we compare it with two different IM selection strategies that are currently used in ACM systems. Either the default IMs (corresponding to a linear channel) are selected, or a user interface based on expert knowledge is used to select the IMs for the channel.

### 6.1. Without Fading

**Figure 7:**
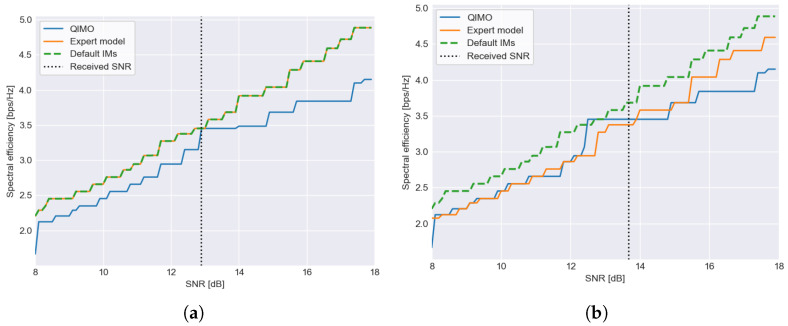
The dependence of QIMO on the received SNR illustrated. The plots show the spectral efficiency for a range of SNR values. QIMO only received data for SNR at the vertical dotted line. (**a**) is the linear channel scenario (scenario 6) and shows that some SNR values do not allow interpolation to lower or higher SNR values. (**b**) is the nonlinear channel (scenario 13) that shows that a slightly different received SNR can allow for interpolation of IM to other (lower) SNR values.

What is important to note is that QIMO is dependent on the received range of SNR values to learn the IMs. This is illustrated in Figure 7, where the efficiency of the selected MODCOD for SNR values between 8 and 16 dB is shown, while there is no fading in both a linear (scenario 6), as shown in Figure 7a, and nonlinear (scenario 13) channel, as shown in Figure 7b, where the expert model is configured correctly. The received SNR value is indicated on the figure as well. In Figure 7a, we can clearly see that the algorithm has only optimized the IMs close to the received SNR values. The efficiency of the IMs selected by QIMO is lower and above and below the received SNR. Only at the received SNR is the efficiency equal to the default IMs and expert model. In Figure 7b, the range of optimized IMs is larger. This difference is due to the value of the received SNR value, which, in this case, is around 13.7 dB, while in Figure 7a, it is 12.9 dB. Receiving data around 13.7 dB allows the algorithm to optimize the IMs of MODCODs in the 16apsk-s2, 16apsk-s2x and 32apsk-s2 clusters, which allows extrapolation to all other 16apsk-s2,16apsk-s2x, and 32apsk-s2 MODCODs. This explains the comparable performance from 8 to 13.5 dB (where the 16apsk-s2 and 16apsk-s2x clusters are located). Because the IM of MODCODs for clusters with higher QEF thresholds (64apsk, 128apsk and 256apsk) cannot be optimized based on SNR values around 13.7 dB, the efficiency of QIMO is lower for SNR values from 14 to 18 dB. Receiving data around 12.9 dB allows the optimization of one MODCOD in the 32apsk-s2 cluster; this allows the algorithm to lower the IMs for the other MODCODs in the cluster. However, due to only receiving data for one MODCOD, this is not enough for optimizing the IMs close to the linear IMs. No MODCODs in the 16apsk-s2 or 16apsk-s2x clusters can be optimized based on this SNR value, which means that the cluster remains at their “safe” margins. This explains the poorer efficiency from 8 to 13 dB.

### 6.2. With Fading

**Figure 8:**
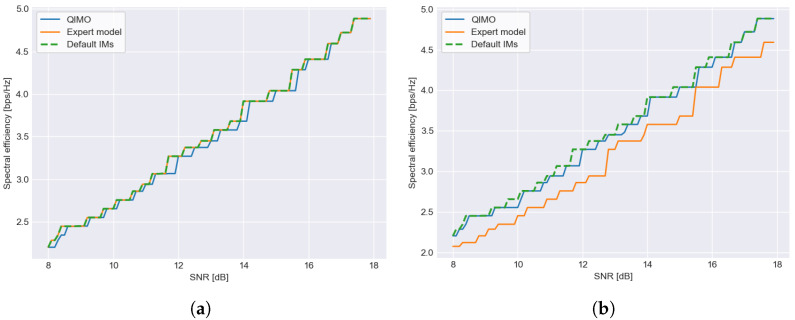
Comparison of spectral efficiency for different IM selection strategies where a range of SNR values (8–18 dB) is received. (**a**) shows the results for the linear channel scenario where the expert model is correctly configured for a linear channel, which means it follows the default IMs exactly (and thus not visible on the figure), and (**b**) shows the results for the same linear channel, but the expert made a mistake and configured IMs for a nonlinear channel. QIMO is close to the default margins and clearly outperforms the expert when it is configured wrongly (**b**).

In Figure 8, the spectral efficiency of the selected MODCOD for SNR values between 8 and 18 dB is shown for the common scenarios when an actual linear channel is encountered without additional effects, the expert is configured correctly (scenario 2) (Figure 8a)) or incorrectly (scenario 1), as shown in Figure 8b. Because the channel is linear, the default IMs are correct in both cases.

We can see in both figures that QIMO learns IMs that are close to the default IMs, as the efficiency curve is close to that of the default. In Figure 8a, the expert is correctly configured to the default IMs. In Figure 8b, the efficiency of the expert model is visibly lower than QIMO and the default IMs, showing that the expert configuring the IMs made a mistake and configured them for a nonlinear channel.

### 6.3. Scenario Evaluation

**Figure 9:**
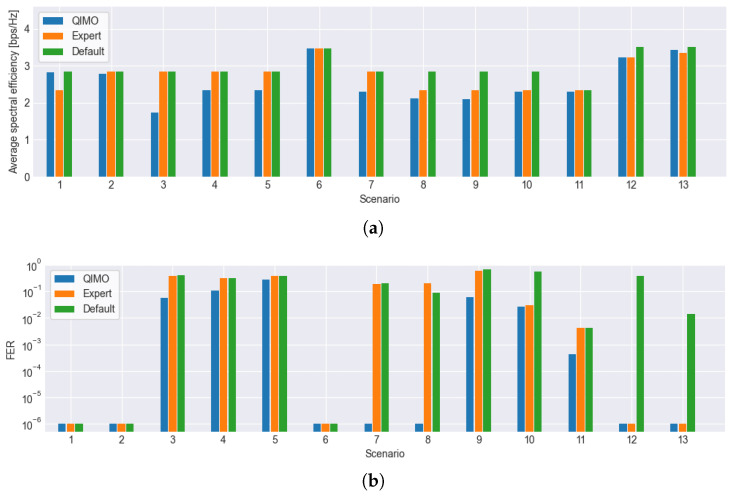
Comparison of average spectral efficiency (**a**) and FER (lower is better) (**b**) of the different IM selection strategies, showing that QIMO learns to lower the average efficiency (by increasing the IMs) to have lower FER than the other IM selection strategies. This is especially visible in scenario 7, where QIMO has the lowest efficiency but much lower FER.

Figure 9 displays the evaluation results for all scenarios listed in Table 4 and all selection strategies. Specifically, Figure 9a shows the average spectral efficiency of the selected IMs, while Figure 9b presents the accompanying FER values. Most notable in Figure 9b is that the FER of QIMO is always lower than or equal to the FER values of the other strategies, showing that it tries to minimize FER and, thus, results in lower FER values than fixed IM strategies. These lower FER values are enabled by increasing the IMs and thus decreasing the average spectral efficiency; this is visible in Figure 9a, as the average spectral efficiency of QIMO is lower in several scenarios. Important to note is that lower average spectral efficiency does not directly mean lower performance, because performance is determined by the frames that actually arrive at the receiver. Transmitting more frames (higher spectral efficiency) with a higher FER can lead to lower overall frames received. This can be illustrated using scenario 7. In this scenario, the actual channel is nonlinear, while a mistake was made using the expert IM selection strategy, causing the expert to use IMs for a linear channel (same IMs as default). This yields higher average spectral efficiency than QIMO but a significantly higher FER due to uncompensated nonlinearities (Figure 9b). QIMO correctly learns higher, safer IMs, resulting in a lower FER and superior overall performance *P*, which prioritizes correctly received frames. The required IM increase scales with nonlinearity intensity (e.g., stronger saturation in Scenarios 10/12), where QIMO adapts automatically and avoids overgeneralization. Scenarios with additional effects negatively influencing the channel (3, 4, 5, 8, 9, and 10) show that QIMO adapts by increasing the IMs (lower spectral efficiency) to try and keep the FER below the desired FER threshold. This is not always successful but leads to lower FER values than the other strategies and to overall performance that should be equal or better than the other strategies. Specifically, in dynamic scenarios with sudden interference/saturation/SNR shifts (3, 4, 8, and 9), the re-convergence signal resets the Q-table to safe margins upon unexpected high FER detection. The fast-convergence phase (low N) enables restoring stability within the accumulation window. Post-adaptation efficiency matches baselines with a significantly lower FER, supporting real-world applicability.

### 6.4. Overall Performance

**Figure 10:**
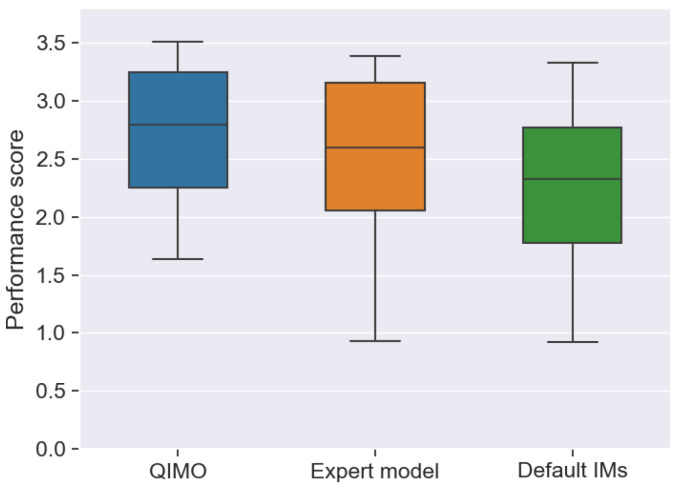
Comparison of performance score (that includes both FER and spectral efficiency) for the three different IM selection strategies in all test scenarios, showing that the overall QIMO performs the best and has less poor performance scores.

To give an overall view of the performance of QIMO, Figure 10 shows the distribution of the performance scores visually for each IM selection strategy. The box represents the interquartile range (IQR) and the difference between the 75th percentile (Q3) and the 25th percentile (Q1), with the whiskers indicating the minimum and maximum values within 1.5 times the IQR. As shown in the figure, the mean of QIMO is above that of the default and expert IMs; this suggests that QIMO has a higher average efficiency. The most notable difference shown in the figure is that the lowest whisker of QIMO is clearly higher, and the distance between the maximum and minimum whiskers is smaller. QIMO therefore exhibits a higher efficiency range with fewer low-efficiency test scenarios and more high-efficiency test scenarios, as the algorithm automatically balances safety margins against performance under varying nonlinearity intensity. This demonstrates that, in addition to the ease of use and eliminating the need for experts, the algorithm has more robust performance with fewer poor performance occurrences.

## 7. Conclusions

The limitations of the current methods of selecting static IMs for ACM in satellite communication systems warrant the development of an optimization algorithm that can adaptively select these IMs for each MODCOD while maximizing throughput, reducing frame errors, and minimizing the need for human input. To address these challenges, a Q-learning-based algorithm was proposed as a suitable solution due to its low computational complexity, fast convergence time, and adaptability in cognitive communication applications. To enable this optimization process without violating the constraint on the FER of user data, the use of “fill frames” was necessary. These frames with dummy data, which are inserted when the (virtual) carrier is incomplete, allow for frame errors without impacting the user frames, thus providing the necessary data for the algorithm. The results demonstrate the effectiveness of QIMO in various scenarios, highlighting its ability to adapt and optimize IMs based on the received SNR values. QIMO consistently exhibits lower FER values compared to other strategies, indicating its focus on minimizing FER and maintaining overall performance. The overall performance of QIMO shows a higher average efficiency compared to both default and expert IMs. Moreover, QIMO displays a higher efficiency range with fewer low-efficiency test scenarios and more high-efficiency test scenarios, emphasizing its robust performance and adaptability. This suggests that QIMO not only offers ease of use and eliminates the need for experts but also provides more consistent and reliable performance across various conditions.

## Figures and Tables

**Table 1:** Summary of related work on margins in ACM.

Paper	Adaptation Type	Adaptation Input	MODCOD- Specific	Compared With
Cione et al. [4]	Static	/	No	/
Ebert et al. [5]	Static	/	No	/
Bischl et al. [6]	Reactive	User Data FER	No	DVB-S2X default fixed margins
K.-M. Ekerete et al. [7]	Reactive	SNR Slope	No	DVB-S2X default fixed margins Bischl et al. [6]
Rico-Alvariño et al. [8]	Adaptive	User Data FER	No	DVB-S2X default fixed margin
Jalali et al. [9]	Adaptive	SNR & Channel Power		Ideal system
Kourogiorgas et al. [10]	Adaptive	SNR Forecast	No	Ideal system
Ferreira et al. [11]	Adaptive	Channel State & PHY/MAC data	No	/
This paper	Adaptive	Fill Frame FER (No user data)	Yes	DVB-S2X default fixed margins Expert margins

**Table 2:** Example of received statistics list by the learning algorithm at each time step.

MODCOD Name	#Frames	#Errored Frames	SNR Estimate
MODCOD 1	136	0	12.2
MODCOD 2	4	0	12.2
…	…	…	…
MODCOD k	4	4	12.2

**Table 3:** Indication of available clusters within a modulation.

	Q PSK	8 PSK	16 APSK	32 APSK	64 APSK	128 APSK	256 APSK
S2	✓	✓	✓	✓			
S2X NL	✓	✓	✓	✓	✓	✓	✓
S2X L		✓	✓	✓	✓		✓

**Table 4:** Overview of test scenarios to evaluate the QIMO compared to the expert and default models.

Scenario Number	Channel Type	Fading (dB)	Expert Config	Dynamic Channel	Additional Effects	Nonlinearity			
						IBO (dB)	SSG (dB)	AM PM (°)	Channel Compression
1	Linear	10–18	Nonlinear	No	/	NA			
2			Linear		/	NA			
3				Yes	Sudden sweeping pure carrier interference	NA			
4					Sudden SNR change to fade 7–15 dB	NA			
5				No	Sweeping purecarrier interference	NA			
6		No	Linear	No	/	NA			
7	Nonlinear	10–18	Linear	No	/	4.5	2	5	Weak/medium
8			Nonlinear	Yes	Slow saturation change (0.1 dB/s)	4.5 to 3	2	5	Weak/medium
9					Sudden saturation change	4.5 to 3	2	5	Weak/medium
10				No	Too much saturation	4.5	2.7	5	Strong
11				No	/	4.5	2	5	Weak/medium
12		No	Nonlinear	No	Too much saturation	4.5	2.7	5	Strong
13				No	/	4.5	2	10	Weak

## Data Availability

The data presented in this study are available on request from the corresponding author.

## Abbreviations

The following abbreviations are used in this manuscript:

FER	Total frame error ratio
$FER_{m}$	Frame error ratio for MODCOD m
P	Performance
M	Set of all available MODCODs
k	Number of available MODCODs
$\eta_{m}$	Spectral efficiency of MODCOD m (bps/Hz)
$IM_{m}$	IM of MODCOD m
$QEF_{m}$	QEF threshold of MODCOD m
$FER_{max}$	The maximal allowed FER on user data
